# Assessing the ability of novel ecosystems to support animal wildlife through analysis of diurnal raptor territoriality

**DOI:** 10.1371/journal.pone.0205799

**Published:** 2018-10-16

**Authors:** Sara Martínez-Hesterkamp, Salvador Rebollo, Lorenzo Pérez-Camacho, Gonzalo García-Salgado, José Manuel Fernández-Pereira

**Affiliations:** Ecology and Forest Restoration Group, Department of Life Sciences, University of Alcalá, University Campus, Alcalá de Henares, Madrid, Spain; Technical University in Zvolen, SLOVAKIA

## Abstract

Novel ecosystems have emerged through human intervention and are rapidly expanding around the world. Whether they can support animal wildlife has generated considerable controversy. Here we developed a new approach to evaluate the ability of a novel forest ecosystem, dominated by the exotic tree species *Eucalyptus globulus*, to support animal wildlife in the medium and long term. To evaluate this ability, we took advantage of the fact that species territory size decreases with increasing habitat quality, and we used territoriality of a raptor guild composed of Northern Goshawk (*Accipiter gentilis*), Eurasian Sparrowhawk (*A*. *nisus*) and Common Buzzard (*Buteo buteo*) as indicator. We compared the territoriality of these species in the novel ecosystem with that in other ecosystems found in the literature. Average distances between con-specifics in the novel ecosystem were similar, or even shorter, than those in other ecosystems. Average distances between Goshawk con-specifics were among the shortest described in the literature. All three species nested preferably in mixed stands abundant in large exotic trees, with high structural complexity and abundance of native species within the stand. Key factors supporting this diverse and dense raptor community were the special forest management system implemented in the study area and the agricultural matrix located close to forest plantations that complements the supply of prey. Our results suggest that forest management that promotes a complex and suitable forest structure can increase the ability of novel forest ecosystems to support wildlife biodiversity, particularly a diverse nesting community of forest-dwelling raptors and their preys. The results further suggest the suitability of territoriality for assessing this potential of novel ecosystems.

## Introduction

One of the major challenges to conservation is biodiversity loss due to habitat alteration or loss [1, 2]. A counter-trend to this habitat loss, which therefore presents intriguing potential for conservation efforts, is the growing presence of novel ecosystems, defined as ecosystems created deliberately or inadvertently by human activity that show a species composition and abundance previously unknown in that area, but do not depend on continued human intervention for their maintenance [3]. Examples of novel ecosystems include forest plantations containing exotic tree species, which account for a quarter of the approximately 264 million ha of forest plantations around the world. This coverage corresponds to 7% of the total global forest area, and it is growing at 5 million ha per year [4]. There is considerable debate about whether these novel ecosystems are useful for biodiversity conservation [3, 5, 6], highlighting the need for rigorous field studies.

Several studies have shown that forest plantations can provide suitable habitats for wildlife (e.g., [5, 7–9]), especially when the afforestations (forest plantations on previous non-forested areas) are compared with adjacent open agricultural areas [10]. In this sense, novel ecosystems can also provide structural heterogeneity at a landscape scale that may help species to exploit resources that would not be available otherwise. However, it would be preferable to study whether novel forest ecosystems can provide suitable habitats for wildlife compared with more natural or less altered forest systems. The possibilities that a novel ecosystem fulfils beneficial functions for wildlife will depend on local conditions and plantation management [11]. A novel ecosystem is more likely to provide these beneficial functions if its structure and resources are similar to those of the native habitat, or if it provides new resources that wildlife can exploit [12].

Our understanding of the ability of novel forest ecosystems to support animal wildlife is hampered by conventional approaches to measuring such potential. Typically, researchers perform a census of the species in the novel ecosystem in order to assess its suitability as a habitat for wildlife [8, 10, 13]. This approach fails to address whether the species in question actually can reproduce successfully within the novel ecosystem [9], which is a key consideration in sustainable biodiversity programs. At the same time, current methods of comprehensively monitoring wildlife populations require significant time, effort and funding [14]. Therefore, it would be useful to find monitoring methods based on certain indicator taxocenosis that assess actual habitat use for reproduction, that report on population persistence in the medium and long term, and that inform about the general condition of fauna and their ecosystem.

One such method may be to analyse the ability of novel ecosystems to support breeding populations of top predators, such as raptors. These species usually require large, high-quality habitats to ensure the presence of sufficient resources [15–17], making them sensitive indicators of habitat suitability for animal wildlife. Indeed, the high position of top predators in food webs makes them sensitive to changes that occur in any part of the network, providing a more integrated view of the ecosystem’s functioning [16, 18]. Several authors have used analysis of raptors’ presence and their habitat preferences as indicators of habitat condition in human-altered ecosystems [7, 19, 20]. In addition, the presence of a diverse community of raptors has been related to high abundance of their prey and potentially with high biodiversity [21, 22].

Simply analysing the presence of predators provides limited information about the suitability of ecosystems for supporting animal wildlife. It may be possible to gain much more detailed insights by analysing the territoriality of predators. Territorial behaviour imposes a minimum distance between con-specifics, which varies according to resource abundance, such that territory size increases when resource abundance is low and decreases when it is high, as it has been demonstrated for different taxa [16, 23–25]. This suggests that nearest neighbour distance (NND) between con-specifics may be useful as an indicator of habitat condition. In our study area, we found that Goshawk´s NNDs increased as the forest structure departed from the preferred breeding habitats characteristics [26]. Analysing territoriality through NNDs and spatial distribution may also provide more detailed information than breeding pair density about how species use the habitat. For example, regular spatial patterns of nests can indicate uniform resource distribution in the habitat [27, 28]. NNDs and spatial distribution may also be more sensitive to habitat changes than breeding pair density. For instance, two areas differing in the abundance and spatial distribution of resources, perhaps due to forest fragmentation or spatial heterogeneity, will differ in average raptor NND and perhaps also in raptor spatial pattern, even though they may support the same density of breeding pairs (e.g., [28–31]). Thus, Demerdzhiev et al. [31] found a similar breeding pair density in two habitats (1 pair/100 km^2^) but a shorter average NNDs in plain areas (9.5 km ±0.67) than in hilly and low mountain areas (10.36 km ±0.86).

The present study analysed the intra-specific territorial behaviour and habitat preferences of a predator guild comprising Northern Goshawk (*Accipiter gentilis*), Eurasian Sparrowhawk (*A*. *nisus*) and Common Buzzard (*Buteo buteo*) in a novel ecosystem. The novel ecosystem is composed mainly of afforestations and naturally regenerated areas containing the exotic eucalyptus tree (*Eucalyptus globulus*) from Australia. First, we compared the observed NNDs with the simulated NNDs obtained with a null spatial model in order to detect whether active nests were closer together or farther apart than predicted by chance (without territorial behaviour). Whether the observed distances are higher than the simulated distances (distances expected by chance), this is the best indicator of existence of territoriality. We also analysed the spatial distributions of the species with G-statistic. Second, in order to assess the ability of this novel ecosystem to provide a suitable habitat for raptors, we compared the observed NNDs with the NNDs from other forest ecosystems reported in the literature. We expected that NNDs would be longer in the novel forest ecosystem than in other forest ecosystems. Finally, we studied the habitat preferences of the three raptors for forest types with different abundance and characteristics of exotic and native tree components in the study area.

## Material and methods

### Study area

The study was conducted on the peninsula of Morrazo (183.3 km^2^) in north-western Spain (Lat 42° 20´ Long 8° 47´) (S1 Fig). The area has a steep topography; elevation ranges from 0 to 625 m a. s. l. (average, 164 m). The climate is humid oceanic with an average annual precipitation of 1,586 mm and average annual temperature of 14.4 °C, allowing high forest production [32]. The potential vegetation of the area is oak wood (*Quercus robur*), with holly (*Ilex aquifolium*), laurel (*Laurus nobilis*) and cork oak (*Quercus suber*) [33]. The forests were replaced by pastures and fields several centuries ago, but reforestation began in the 16th century, reaching its peak around 1940, when extensive plantations of eucalyptus (*Eucalyptus spp*) were created [34].

Nowadays nearly half the area (42.5%) is covered by pure eucalyptus forests *(Eucalyptus globulus*) or mixed forest of eucalyptus with English oaks (*Q*. *robur*) and maritime pines (*Pinus pinaster*). Young tree formations cover 8.4% of the area; crops and scattered houses, 35.5%; and urban areas, scrublands and coastal habitats, 13.6%. Some patches of eucalyptus forest remain planted and managed, but in many cases their expansion occurs spontaneously [34]. The forests are mainly private, with low-intensity harvests occurring in small plots, 80% of which are smaller than 0.5 ha [32]. Rotation age varies, with some sites undergoing rotations exceeding 50 years. This high fragmentation and variety in rotations has led to a mosaic of stands showing substantial heterogeneity in maturity, tree structure, abundance of exotic and native tree components, vegetation height and abundance of large trees (S1 Table).

Since eucalyptus is present in all types of forest in the study area and in fact dominates the forests, we considered the entire forest system as a novel ecosystem. The landscape-scale habitat is heterogeneous, arranged in a mosaic of forest and non-forest areas (S2 Fig).

### Species analysed

Goshawks are medium-sized raptors (weight range, 814–1,510 g) and Sparrowhawks are small raptors (151–268 g) [35]. Both raptors show territoriality during the breeding season, frequently leading to regular spatial nest distribution [28, 36, 37]. Buzzards are medium-sized raptors (804–923 g; [35]) that, unlike the other two species, show differences in territorial behaviour across populations. Populations range from being regularly spaced with clearly defined territories, to having pairs that cluster together, with small nesting areas and communal undefended hunting areas [16]. Several authors have noted that the nesting territory of these species increases as their preys become less abundant or accessible [38–40]. The three native species show similar nesting habitat requirements, though they appear to show some differences in topography and forest structure preference. For example, Fasola and Zanghellini [41] reported elevational differences among these species, and Newton [42] reported that Sparrowhawks use younger forest stands than Goshawks. The three species show some overlap in diet, with Goshawks feeding on medium-sized birds and mammals, Sparrowhawks on small birds [42–46] and Buzzards on small and medium-sized mammals and reptiles [47, 48]. More than 70 species of birds, mammals and reptiles were consumed by the three raptor species in the studied area (see S2 Table).

All the work was conducted in accordance with relevant national and international guidelines and conforms to the legal requirements of the regional government (Dirección Xeral de Conservación da Natureza of the Xunta de Galicia) which granted permission to carry out the study.

### Estimation of territorial parameters and habitat preferences

The forest patches were systematically surveyed on foot for Goshawk and Buzzard nests for eight years (2004–2011) and for Sparrowhawk nests for six years (2006–2011) in order to locate the active nests. Nests were visually searched in any season or after detecting the presence of raptors (adult, nestling or fledgling), auditory and visually, during the breeding season (playback was not used). All the registered nests were checked for raptor activity during the breeding season. The relatively small size of the study area and the species’ site fidelity and nest reuse facilitated detection of active nests. Four to six proficient surveyors participated in the surveys each year. Detection probability of active nests is supposed to be more than 90% per year although no detection protocol was used.

We mapped and quantified the different forest types in the study area (S1 Table and S2 Fig). Forest stand composition and structure were determined based on photo-interpretation of satellite images of the year 2009 published by PNOA (Plan Nacional de Ortografía Aérea http://pnoa.ign.es/). Forest types were digitized using a geographic information system (ArcGIS 10, [49]) and confirmed with field visits.

To analyse intra-specific distances, we estimated NNDs, defined as the distance between the active nest of a species and the nearest active nest of the same species. Active nests were those where incubation was observed [50]. We also estimated the NND expected by chance for each active nest. Using a null spatial model, we measured the NND between each active nest of a species and the nearest simulated “nest” of the same species located at random. For each year and species, 100 simulated sets of randomly distributed “nests” were used, with each set featuring a number of random “nests” equal to the number of active nests for that particular species in that particular year minus one nest. For example, for Goshawk in 2004 (22 active nests), we simulated 100 iterations of 21 random “nests”, and for each iteration we estimated the NND of each of the 22 active nests to the random “nests”. The null spatial model took into account the habitat preferences of each species in the following three ways. First, the proportions of simulated nests in each forest type matched those observed in the study area (Table 1). Second, forest patches smaller than the smallest patch used by a given species were removed (Goshawk and Sparrowhawk, 4.1 ha; Buzzard, 6.0 ha). Third, elevations above the highest elevation where nests were found were also removed (Goshawk, 340 m; Sparrowhawk and Buzzard, 370 m).

**Table 1:** Proportion of observed nests of the three raptor species found in each forest type of the study area (S1 Table and S2 Fig), and use preferences of each forest type in relation to the proportion of forest area available (Ivlev’s index).

		Proportion (%)				Ivlev’s index^b^		
	Forest type^a^	Goshawk nests	Sparrowhawk nests	Buzzard nests	Forest Area	Goshawk preferences	Sparrowhawk preferences	Buzzard preferences
1	**Old mixed Eucalyptus stands**	39.2	47.6	43.8	21.0	0.30*	0.39*	0.35*
2	**Mixed Eucalyptus stands**	43.1	39.6	40.8	23.9	0.29*	0.25*	0.26*
3	**Burned Eucalyptus stands**	5.9	5.5	4.6	4.1	0.18*	0.15*	0.06*
4	**Monospecific Eucalyptus stands**	8.5	3.7	4.2	12.2	-0.18	-0.53	-0.49
5	**Forests with scattered trees**	0.0	0.0	1.2	3.2	-1.00	-1.00	-0.45
6	**Deciduous riverbank forests**	0.0	0.0	0.0	1.7	-1.00	-1.00	-1.00
7	**Oak, chestnut and cork oak woods**	2.0	0.0	1.5	4.7	-0.40	-1.00	-0.52
8	**Pine forests**	0.0	0.6	0.0	5.0	-1.00	-0.79	-1.00
9	**Burned pine forests**	0.0	0.0	0.0	0.2	-1.00	-1.00	-1.00
10	**Acacia forests**	0.0	1.8	0.0	0.4	-1.00	0.64*	-1.00
11	**Young plantations**	0.7	1.2	3.5	16.6	-0.92	-0.87	-0.65
12	**Recently logged forests**	0.7	0.0	0.4	7.1	-0.82	-1.00	-0.89
	**TOTAL**	100	100	100	100			

^a^ For a complete description of forest type composition and structure, see S1 Table.

^b^ The * denotes a positive value of Ivlev’s index, indicating that the forest type is preferred, whereas negative values indicate that the forest type is used below its availability.

Observed and simulated NNDs were estimated both annually (active nests) and multi-annually (nesting territories over the entire study period). This allowed us to compare our results with data for many other populations of the studied species, since both approaches are frequently used in the literature (e.g., [28, 30, 37, 48]). Multi-annual analysis included data from all nesting territories active for at least one year during the study period. Each nesting territory was represented by a centroid or set of average coordinates computed from the position and frequency of use of all active nests within that territory over the entire study period. A geographic information system (ArcGIS 10.0, [49]) was used to generate the randomly distributed simulated nests and calculate NNDs.

We estimated the average density of breeding pairs annually and multi-annually in the study area from the number of active nests each year and the number of active nesting territories over the entire study period, respectively. To test whether the number of breeding pairs increased or decreased over the years, we performed linear correlations between the number of active nests of each species and the year of study. To analyse whether observed NNDs varied over the years, average NNDs for each year were compared using one-way ANOVA. These analyses were performed using Statistica 8.0 [51].

We used two approaches to compare observed and simulated NNDs. In the first approach, we used generalized linear mixed models (GLMMs) to detect whether the average distances between nests for each species were larger or smaller than simulated distances, in order to detect whether con-specific nests were closer together or farther apart than predicted by chance. For each species we fitted a gamma GLMM with a long-link function, where the response variable was the NND, the fixed factor was the type of NNDs (observed/simulated), and the random factors were the year, territory and nest (from now on, we will refer to this as “full model”), see a similar procedure in Rebollo et al. [52]. The random factors allowed us to control for spatiotemporal dependence of the data. Each of these models was compared with its null model, which did not include the fixed factor (type of NNDs). We assessed model fit using the Akaike Information Criterion modified for small sample sizes (*AICc*). If the *AICc* of the full model was smaller than that of the null model and the difference in *AICc* values (*Δ*_*i*_) was at least 6 points [53], we concluded that the full model was more plausible than the null model. In other words, the observed distances were different from those expected by chance. GLMMs were calculated using the "lme4" package [54] in R 3.0.1 [55].

In the second approach, we compared frequency bar graphs of observed distances and of randomly distributed simulated distances. Our goal was to detect ranges (bins) of distances from which pairs were excluded (*i*.*e*., unable to nest) and ranges of distances from which the proportions of pairs were substantially smaller or larger than expected by chance. Frequencies of observed and simulated distances were estimated separately for each year, and the mean frequency and SE were calculated over the entire study period.

For each species we calculated the G index [56], which estimated the spatial distribution of the territories; indices from 0 to 0.65 indicate random distribution, whereas values above 0.65 indicate increasing regularity. This index has been used in numerous studies to analyse patterns of raptor spatial distribution and infer territorial behaviour (e.g., [28, 37, 48]).

The average NND among active nests (annual scale) and the average NND between territories (multi-annual scale) for each species were compared with distances reported in other populations of the same species (see S3 Table). I.e., once the NNDs data from bibliography were arranged in ascending order, we positioned our NNDs results, reporting the position, as a percentage, where our results in the group fall (percentile). For example, the 20th percentile is the value under which 20% of the observations from the literature are, i.e. 20% of the shorter NNDs. In the case of Goshawk, we compared our results only with data from Palearctic populations (i.e., subspecies *A*. *gentilis gentilis*).

Finally we estimated the preference of each species for different forest types using the common Ivlev´s electivity index [57] that allows us to compare data in terms of percentage:

$$S=\frac{\left(r-p\right)}{\left(r+p\right)}$$

where *r* represents the percentage of nests found in a forest type and *p* represents the availability of that forest type (percentage of total forest area occupied by that forest). This index ranges from -1 to +1, and positive values were considered to indicate preference for a given forest type.

## Results

**Fig 1:**
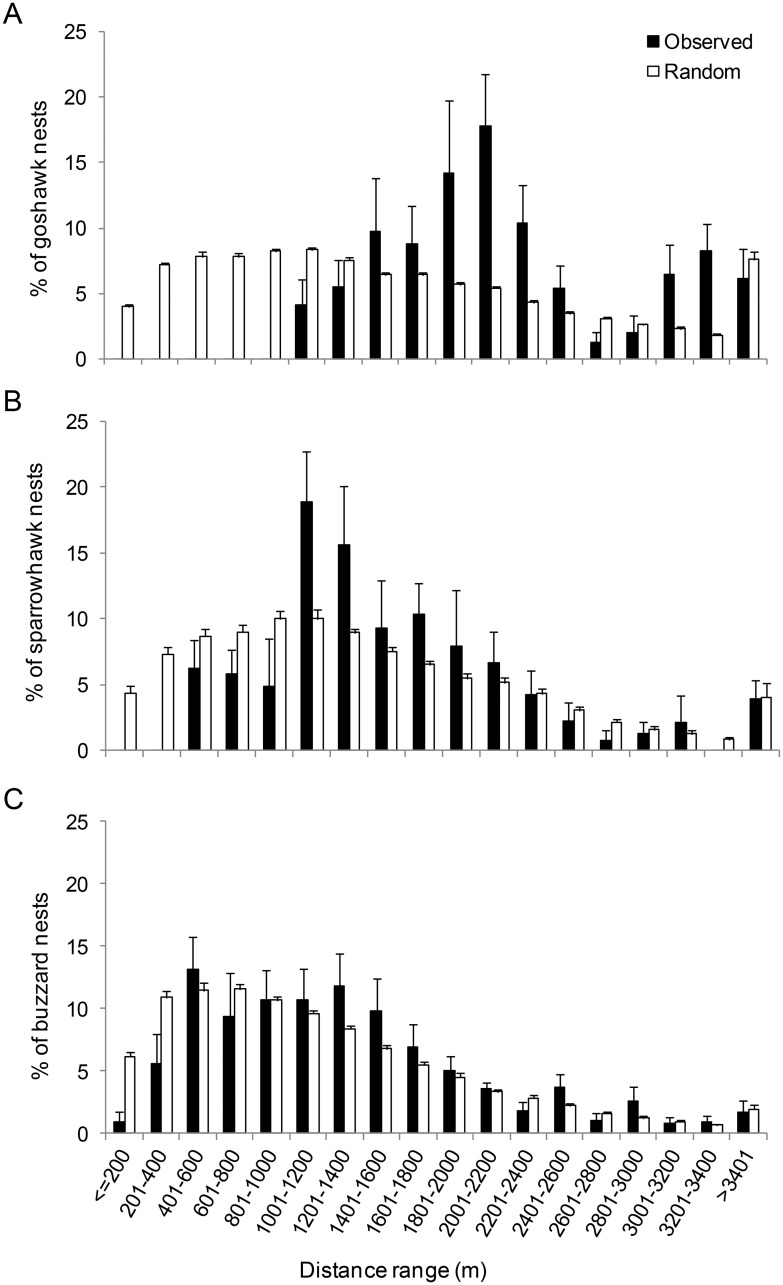
Frequency distribution of distances among nests. Both observed (black bars) and expected by chance based on the null model (white bars) are showed for (A) Goshawk, (B) Sparrowhawk and (C) Buzzard. Bar graphs show the mean percentage of nests per year located within each distance range (bin). Error bars indicate standard error. Originally drawn by the authors.

Goshawks showed the longest NNDs between nests (mean [SE]: 2,234.1 m [162.1]), followed by Sparrowhawks (1,567.8 m [153]) and Buzzards (1,323.7 m [138.7]) (Table 2). Mean NNDs did not vary significantly over the study period for Goshawks or Sparrowhawks, but they did for Buzzards (*F*_(7,252)_ = 2.78, *p* = 0.008). Observed distances between nests were greater than distances expected by chance in all three species, both annually and multi-annually (Table 3), indicating that territorial behaviour was influencing the spatial distribution of the three species. The minimum distance between active nests during the study period was 1,034 m for Goshawks, 467 m for Sparrowhawks and 178 m for Buzzards (Table 2). Among Goshawk nests, distances shorter than 1,500 m were less frequent than would be expected by chance (Fig 1). Among Sparrowhawk and Buzzard nests, distances below 1,000 m and 400 m, respectively, were less frequent than would be expected by chance.

**Table 2:** Number of active nests per year and nesting territories (n), density of breeding pairs, average nearest neighbour distance (NND) and range of observed distances for each raptor species.

Species	Year/Territories	n	Density (pairs/100 km^2^)	Mean NND ± SE (m)	NND range (m)
**Goshawk**	2004	22	12.0	2,170.8 ± 136.2	1,489.3–3,395.3
	2005	20	10.9	2,305.3 ± 153.8	1,489.3–3,409.4
	2006	20	10.9	2,198.9 ± 122.8	1,534.2–3,234.9
	2007	18	9.8	2,209.0 ± 212.3	1,199.8–4,532.2
	2008	18	9.8	2,404.3 ± 179.8	1,034.4–3,421.8
	2009	18	9.8	2,070.5 ± 132.8	1,300.4–3,439
	2010	18	9.8	2,433.8 ± 216.8	1,399.6–4,590.2
	2011	19	10.4	2,080.2 ± 142.1	1,138.3–3,225.8
	Territories	29	15.8	1,933.3 ± 84.4	1,367.9–3,283.2
**Sparrowhawk**	2006	16	8.7	1,774.3 ± 268.0	546.6–4,651.8
	2007	22	12.0	1,718.8 ± 182.8	584.5–3,921.6
	2008	37	20.2	1,310.3 ± 84.7	693.6–2,556.0
	2009	26	14.2	1,486.2 ± 140.4	542.7–3,528.2
	2010	25	13.6	1,592.4 ± 105.8	611.7–2,855.3
	2011	24	13.1	1,525.1 ± 136.5	467.3–3,604.3
	Territories	57	31.1	1,052.5 ± 55.9	423.6–2,266.9
**Buzzard**	2004	29	15.8	1,259.0 ± 170.3	178.0–3,284.7
	2005	26	14.2	1,591.3 ± 167.1	365.3–4,586.9
	2006	28	15.3	1,657.4 ± 169.6	510.7–4,156.3
	2007	39	21.3	1,082.8 ± 102.1	499.9–3,139.0
	2008	32	17.5	1,494.9 ± 166.1	294.9–4,744.7
	2009	34	18.5	1,295.4 ± 121.8	288.9–2,877.5
	2010	42	22.9	1,137.1 ± 86.8	302.8–2,732.0
	2011	30	16.4	1,071.5 ± 125.8	213.8–2,523.8
	Territories	84	45.8	742.3 ± 38.8	168.4–2,484.8

**Table 3:** Results of generalized linear mixed models (GLMMs) comparing the observed distances with simulated distances expected by chance between active nests and nesting territories (T) for each raptor species.

Species	*AICc*_*0*_	*AICc*_*1*_	*Δ*_*i*_	*α ± SE*	*β ± SE*
**Goshawk**	253,793	253,737	57	1,617.64 ± 54.75	686.05 ± 107.70
**Sparrowhawk**	242,909	242,899	10	1,410.36 ± 61.68	234.38 ± 70.65
**Buzzard**	413,215	413,203	12	1,168.30 ± 32.50	168.85 ± 46.56
**Goshawk T**	46,805	46,788	16	1,248.70 ± 47.71	735.93 ± 228.15
**Sparrowhawk T**	87,259	87,253	6	857.49 ± 22.61	206.71 ± 80.14
**Buzzard T**	123,856	123,849	7	653.84 ± 16.67	132.66 ± 49.45

*AICc*, Akaike Information Criterion modified for small sample sizes; *AICc*_*0*_, AICc for null model; *AICc*_*1*_, AICc for full model; *Δ*_*i*_ = *AICc*_*0*_*—AICc*_*1*_. The full model was considered more plausible than the null model when AICc_1_ was lower than *AICc*_*0*_ by at least 6 points (*Δ*_*i*_ ≥ 6). Coefficients of the full model denote mean distances predicted by the model: *α* (Intercept) = mean of simulated distances predicted by the full model; *β* = mean difference between observed and simulated NNDs.

Goshawks and Sparrowhawks showed a regular spatial pattern annually (Goshawk, G_nests_ = 0.83; Sparrowhawk, G_nests_ = 0.67) and multi-annually (Goshawk, G_territories_ = 0.90; Sparrowhawk, G_territories_ = 0.74). Buzzards showed a regular spatial pattern only multi-annually (G_nests_ = 0.51, G_territories_ = 0.65). Note that observed distances between Buzzard nests were greater than distances expected by chance also annually, indicating that the territorial behaviour influenced the annual spatial distribution of this species, although this did not promote a regular spatial pattern of Buzzards at the annual scale.

**Fig 2:**
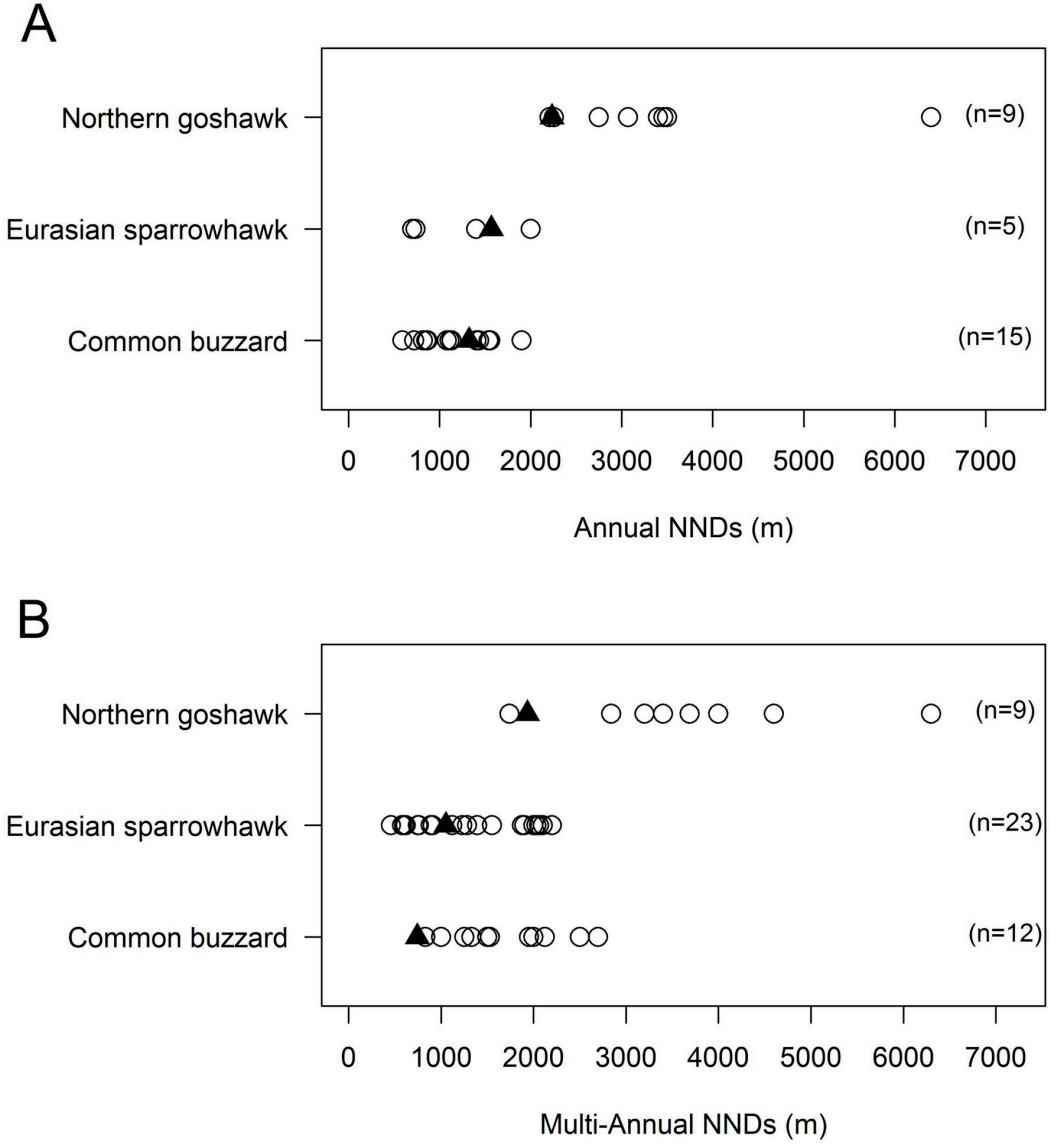
Comparison of mean NNDs of several populations of Goshawks (Palearctic populations only), Sparrowhawks and Buzzards. (A) Mean distance between active nests (annual scale). (B) Mean distance between territories (multi-annual scale). Black triangles indicate NNDs measured in the present study; white circles, NNDs obtained from the literature (see S3 Table for complete data and references). Originally drawn by the authors.

We compared the mean NNDs observed in our study with those reported for other populations of the three species in the literature. Average annual and multi-annual NNDs were shorter for Goshawks than for other Palearctic populations, falling within the 1st and 5th percentiles, respectively, of the range of NNDs described in the literature (Fig 2). For Sparrowhawks, average annual NND ranked in the 67th percentile, while average multi-annual NND ranked in the 34th percentile. Average annual NND of Buzzards fell within the 56th percentile, whereas average multi-annual NND was the shortest of all populations compared.

The three species of raptors preferred to nest in more developed eucalyptus stands, comprising mixed mature eucalyptus, mixed eucalyptus and burned eucalyptus stands (Table 1). These three forest types are those showing greater coverage and abundance of large eucalyptus (S1 Table). The Sparrowhawk also showed positive values of the Ivlev index in stands of Australian blackwood (*Acacia melanoxylon*), another exotic tree in the study area. The eucalyptus tree was the most frequent nest tree: Goshawks 92.2% of the nests (n = 64), Sparrowhawks 53.7% (n = 82), and Buzzards 65.6% (n = 96).

In our study area, the average density (SE) of active breeding pairs per 100 km^2^ each year was in order of decreasing density: 17.7 (1.1) for Buzzards, 13.6 (1.5) for Sparrowhawks and 10.4 (0.3) for Goshawks (Table 2). The number of active Goshawk nests each year tended to decrease during the study period (*n* = 8, *r* = -0.74, *p* = 0.036), while the number of active nests of Buzzards and Sparrowhawks did not change significantly over the study period. The number of nesting territories active during at least one year of the study period per 100 km^2^ was 45.8 for Buzzards, 31.1 for Sparrowhawks, and 15.8 for Goshawks.

## Discussion

Nesting territories of the raptor guild in this novel ecosystem were similar in size or even smaller than those of other populations cited in the literature. This suggests that the novel ecosystem provided a similarly good or even better nesting habitat for these top predators than other ecosystems. Thus, these novel ecosystems may provide suitable reproductive habitats for animal wildlife, if managed appropriately. Additionally, the results of the present work highlight the potential of using territoriality measures of top predators to assess a novel ecosystem’s ability to support animal wildlife.

For Goshawks, both average distance between active nests each year and distance between nesting territories over the study period (2004–2011) were the second shortest of the corresponding distances reported in the literature. This suggests that the study area provided suitable nesting habitats and abundant resources for this species (see also Pérez-Camacho *et al*. [45] and Rebollo *et al*. [46]). The observed average reproductive success (2.3 fledglings per active nest, [26]) is also high in the European context, where the corresponding values are 1.8 in northern, central and western Europe, and 1.6 in southern Europe [58]. In fact, the average distance between active nests was similar over the years, suggesting that the nesting habitat remained suitable throughout the eight years of the study period. We attribute the observed decline in the number of breeding pairs of Goshawks during the study period not to declining habitat suitability but rather to illegal human activities. We observed Goshawks caught in Swedish traps within the study area, likely reflecting the fact that 20% of the Goshawk diet in the study area is domestic prey (S2 Table) [46]. This decline in the Goshawk breeding population did not significantly affect the regular spatial distribution of active breeding pairs during the study period, though the decline may cause measurable effects if it continues. Since the early 1980s, the spatial distribution of Goshawk pairs has remained nearly constant, and many nesting territories have remained in the same place with nests sometimes even in the same trees (unpublished data). These observations suggest that this novel forest ecosystem can provide suitable nesting habitat for a dense Goshawk population in the long term, as long as the decline of breeding pairs by unnatural causes is prevented.

For Sparrowhawks, the average distances between active nests and between nesting territories were similar to the average values reported in the literature, suggesting that the study area provided nesting habitats of average suitability for this species. The spatial distribution pattern was regular, although less regular than that of other Sparrowhawk populations [28]. The presence of a large Goshawk population in the study area may explain why the Sparrowhawk distribution was less regular than in other populations, as Goshawk is a predator of Sparrowhawk [44, 46]. This suggests that the distances between Sparrowhawk nests would be shorter in the absence of Goshawks. Indeed, other evidence supports the idea that Goshawk presence influences the position of Sparrowhawk nests; Sparrowhawks nest farther apart from active Goshawk nests [59] which can decrease the spatial regularity of Sparrowhawk nesting territories [42].

The novel ecosystem also appears to provide a suitable nesting habitat for Buzzards, given that the distance between active nests each year was similar to the average reported in the literature, and the multi-annual distance between territories was the shortest of all populations reported. We attribute the small territory size observed in the present study to good resource conditions, since Buzzards showed territorial behaviour both annually and multi-annually, although their nests showed irregular spatial distribution on the annual scale.

The three species preferred to locate their nests in structurally more complex eucalyptus stands, rather than in remnants dominated by native tree species. Most nests were placed in eucalyptus stands with a high density of large eucalyptus trees and native vegetation, generating a complex vertical structure. These stands provide the raptors with a new structural element that native stands do not, namely, large vertical trees with smooth bark, where raptors preferred to locate their nests (mean height of eucalyptus trees with nests was 35.6 m, SE = 0.7, n = 142 and mean height of non-eucalyptus trees with nests was 20.6 m, SE = 0.6, n = 69; [60]). Raptors likely prefer these stands for their protective potential, since complex forest structure helps conceal the nests from predators and human disturbance [59, 61, 62]. In addition, high trees position the nests out of the reach of land predators that pose a risk to Buzzards and Goshawks, since airborne predators like the Eurasian Eagle-Owl (*Bubo bubo*) was absent from the study area.

The three raptors rejected the younger and monotonous eucalyptus stands that showed less diversity of tree species and greater abundance of coetaneous trees. These stands contain a smaller proportion of large eucalyptus. This may help explain why eucalyptus plantations on the Iberian Peninsula with shorter rotation periods show lower forest raptor densities than the present study area [48, 63]. Instead, raptors on those plantations locate their nests in pines, even though eucalyptus trees are more abundant. For instance, in a population of Buzzards in Bizkaia, where the area is mainly covered by Eucalyptus plantations with short rotations and, to a less extent, with pine plantations, pines were positively selected (92% of nests) while Eucalyptus were negatively selected (7% of nests) [48]. This suggests that shorter rotation periods may not allow adequate structural development of the canopy to favour raptor nesting, and that appropriate management of forest plantations with exotic trees is key to ensuring that novel ecosystems support wildlife [3, 11]. In our study area, the large number of small-sized plots for forest exploitation and the presence of many owners who establish different rotation periods on their plots have led to a mosaic of stands showing different ages, complexities and structures. Indeed, forest exploitation is not carried out on many plots, allowing the appearance of mature stands with large trees and high structural complexity. The characteristics of the eucalyptus tree in the study area and its heterogeneous management meet some of the requirements proposed for managing plantations for biodiversity conservation (see [8]).

Given that territory size probably reflects the abundance, distribution and accessibility of prey within territories (e.g., see references in the introduction and [28, 38, 50, 64], the relatively short distances observed for all three raptor species suggest relatively high prey densities in the study area. This contrasts with the fact that plantations usually contain less abundant and diverse potential prey species than native forests [10], as reported for eucalyptus plantations in another region of northern Spain [65]. In the present study, forest areas lie close to agricultural areas (forests and young tree formations cover 50.9% and agricultural areas cover 35.5%), forming a farmland-forest mosaic where hunting options are likely higher than they would be if only forest prey were available. Indeed, Buzzards often hunt in open areas, so forest prey is less important in their diet [47]. At least half the diet of the Goshawks in the study area comprises prey from non-forest areas, such as the Domestic Pigeon (*Columba livia var domestica*), Eurasian Collared Dove (*Streptopelia decaocto*) and the Yellow-legged Gull (*Larus michahellis*) [46]. In this way, the present novel ecosystem fulfils a structural function, providing suitable habitat to locate the nest and protect offspring within an agricultural matrix that complements the prey range from the forest. These results highlight the importance of landscape-level heterogeneity for some novel ecosystems [8,66]. Novel ecosystems that increase heterogeneity and complement the functions provided by surrounding areas are more likely to support dense populations of top predators.

Other forest diurnal raptors also nest in the present novel ecosystem, such as the Eurasian Hobby (*Falco subbuteo*) and the European Honey Buzzard (*Pernis apivorus*). We did not include these species in the analysis for lack of adequate data about population density and spatial distribution. Their presence in the area provides further, indirect support that this habitat provides suitable nesting characteristics for a diverse guild of forest raptors, including some specialist species. These results are consistent with other studies showing that well-managed forest plantations can support populations of specialist species and of birds of conservation interest (e.g., [5, 7, 12, 67]).

The studied agroforestry system presents a high density of breeding pairs of the three raptor species. For example, with 15.8 nesting territories per 100 km^2^ and 10.4 active pairs per year per 100 km^2^, the breeding density of this Goshawk population during the study period is among the densest in Europe, where it averages 3.4 active pairs per 100 km^2^, with most of the populations below 10 active pairs per 100 km^2^ [43]. This may have some implications of interest for wildlife conservation. Even though it seems to be good for these forest raptors, what may happen with an enhanced predation in places where these novel environments are? Many prey species would be affected and their conservation could be compromised by the new presence of a top predator that should not be there or by a higher abundance of predators. For example, Goshawks exert significant predation pressure on smaller predators, both diurnal and nocturnal, and the increase of Goshawk population may influence their abundance and spatial distribution [68, 69]. Thus, the decrease of the Kestrel (*Falco tinnunculus*) populations in the study area in the last decades may be related to the increase of the forest-dwelling raptors. The increase of raptor density can also alter inter-specific interactions between forest-dwelling raptor species. We analysed this effect in Rebollo et al. [52]. The inter-specific interactions within the raptor guild influenced the spatial distribution of predator species in the forest ecosystem, with intraguild predation as a key driver. In that study, we discussed several mechanisms that may promote the coexistence of subordinate and dominant predators and the spatial assembly of this raptor guild. The high density of raptors is also generating a wildlife-human conflict by predating on domestic prey, especially by Goshawk, the dominant raptor in the study area. If the illegal hunting pressure on Goshawk goes on in the study area, this novel ecosystem might behave as a trap habitat due to an excess of a non-natural mortality produced by humans. In this sense, the rapid decrease of Goshawk populations in the last years in the study area is disturbing. This may not happen in a pristine environment where interaction with humans should be less frequent.

### Final remarks

Whether novel ecosystems should be maintained and managed for conservation purposes has generated considerable debate, but we should not ignore their existence or potential as habitats to support wildlife [6, 13]. The present work suggests that if a novel ecosystem cannot be restored to its original native form, appropriate management can still enable it to serve as a suitable nesting habitat for a diverse, dense community of top predators in the medium to long term. However, in our studied system, it does not seem to be much deliberate, coordinated management in this landscape, but rather resulting the mosaic from the many different owners with different approaches to land management.

At the same time, our findings highlight the importance of complementarity between the novel ecosystem and surrounding areas: the novel ecosystem in the present study provides new structures (tall trees and forest stands with complex vertical structure), which offer a suitable nesting habitat for raptors; while neighbouring ecosystems provide additional resources such as abundant prey, which are required for maintaining the raptor population. In that sense, the entire system could be considered as the novel ecosystem, i.e., the mix of exotic tree stands in an agricultural matrix, not just the tree stands alone.

Our study provides evidence that analysing the territoriality of top predators can serve as a synthetic method for evaluating novel ecosystems and their surroundings, since territory size reflects the quality of the habitat. In addition, analysing guilds of raptors that show relatively small overlap in their diets, as we did in the present work, may indicate the presence of a diverse community of prey in the study area (> 70 prey species in our case). In conclusion, the possibility of evaluating and managing novel ecosystems to maximize their potential for supporting biodiversity can provide more options for wildlife management, especially when funding for conservation projects and restoration to native forests is limited.

## Supporting information

S1 Fig Location of the study area in southwestern Europe. (DOCX)

S2 Fig Map of forest types in the study area. The figure was prepared based on forest stand composition and structure determined from aerial photographs and confirmed with field visits. White areas are non-forest habitats.
(DOCX)

S1 Table Characteristics and description of forest types based on composition and structure. Proportion of total forest area occupied by each forest type, relative and average (± SD) tree height in the forest patch, relative and average abundance of eucalyptus trees, large eucalyptus and large oaks. Average tree height and abundance were measured in 10 m radius circular plots (0.03 ha); *n* = number of sampled plots; *DBH* = Diameter at breast height. For sample details, see García-Salgado et al. (2018)**.
(DOCX)

S2 Table Prey species in the three raptors diets in the study area. Prey species in the Northern Goshawk, Eurasian Sparrowhawk and Common Buzzard diets during the breeding season in the study area. 2,618 prey items of Goshawk were identified from camera images taken at the nests and from uneaten prey remains collected in the nests and surrounding plucking sites during 2008−2011 (77 nests were studied in 29 nesting territories and at least 31 different prey species were hunted). See García-Salgado et al. (2015) and Rebollo et al. (2018) for a complete description of the diet of Goshawk in the area and of the methodology used to study the diet. 1,021 prey items of Sparrowhawk were identified from uneaten prey remains collected at the nests and surrounding plucking sites during 2010–2011 and 2014–2015 (70 nests were studied in 46 nesting territories and at least 51 different prey species were hunted)–unpublished data-. 27 prey items of Buzzard were identified from uneaten prey remains collected at the nests and surrounding plucking sites during 2010–2017 (21 nests were studied in 18 nesting territories and at least 12 different prey species were hunted)–unpublished data-. At least 71 different species of birds, mammals or reptiles were hunted by the three raptor species.
(DOCX)

S3 Table Data and sources for all populations of the three raptors used to prepare Fig 2. Country, population, time scale estimation, number of nests, mean nearest neighbor distances (NND), SD of the mean, pair density, and data sources for all populations of Goshawk, Sparrowhawk and Buzzard used to prepare Fig 2.
(DOCX)
