# Supplementary material for: Assessing the ability of novel ecosystems to support animal wildlife through analysis of diurnal raptor territoriality
Source: PLoS One. 2018 Oct 16;13(10):e0205799. doi: 10.1371/journal.pone.0205799 (PMC6191124; doi:10.1371/journal.pone.0205799)
Supplement: S1 Fig — (DOCX) [file pone.0205799.s001.docx]

**Supporting Information**

**Assessing the ability of novel ecosystems to support animal wildlife through analysis of diurnal raptor territoriality**

S. Martínez-Hesterkamp, S. Rebollo, L. Pérez-Camacho, G. García-Salgado and J.M. Fernández-Pereira


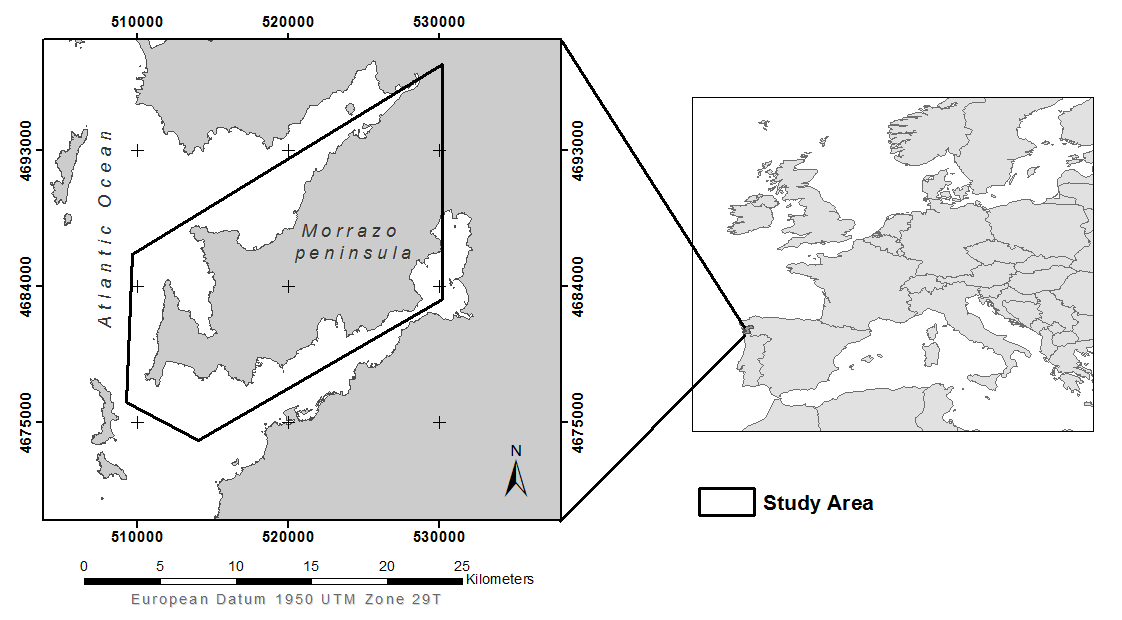


**S1 Figure**. Location of the study area in southwestern Europe, on the northwestern part of the Iberian Peninsula. Originally drawn by the authors.
