# Supplementary material for: Assessing the ability of novel ecosystems to support animal wildlife through analysis of diurnal raptor territoriality
Source: PLoS One. 2018 Oct 16;13(10):e0205799. doi: 10.1371/journal.pone.0205799 (PMC6191124; doi:10.1371/journal.pone.0205799)
Supplement: S2 Fig — The figure was prepared based on forest stand composition and structure determined from aerial photographs and confirmed with field visits. White areas are non-forest habitats. (DOCX) [file pone.0205799.s002.docx]

**Supporting Information**

**Assessing the ability of novel ecosystems to support animal wildlife through analysis of diurnal raptor territoriality**

S. Martínez-Hesterkamp, S. Rebollo, L. Pérez-Camacho, G. García-Salgado and J.M. Fernández-Pereira


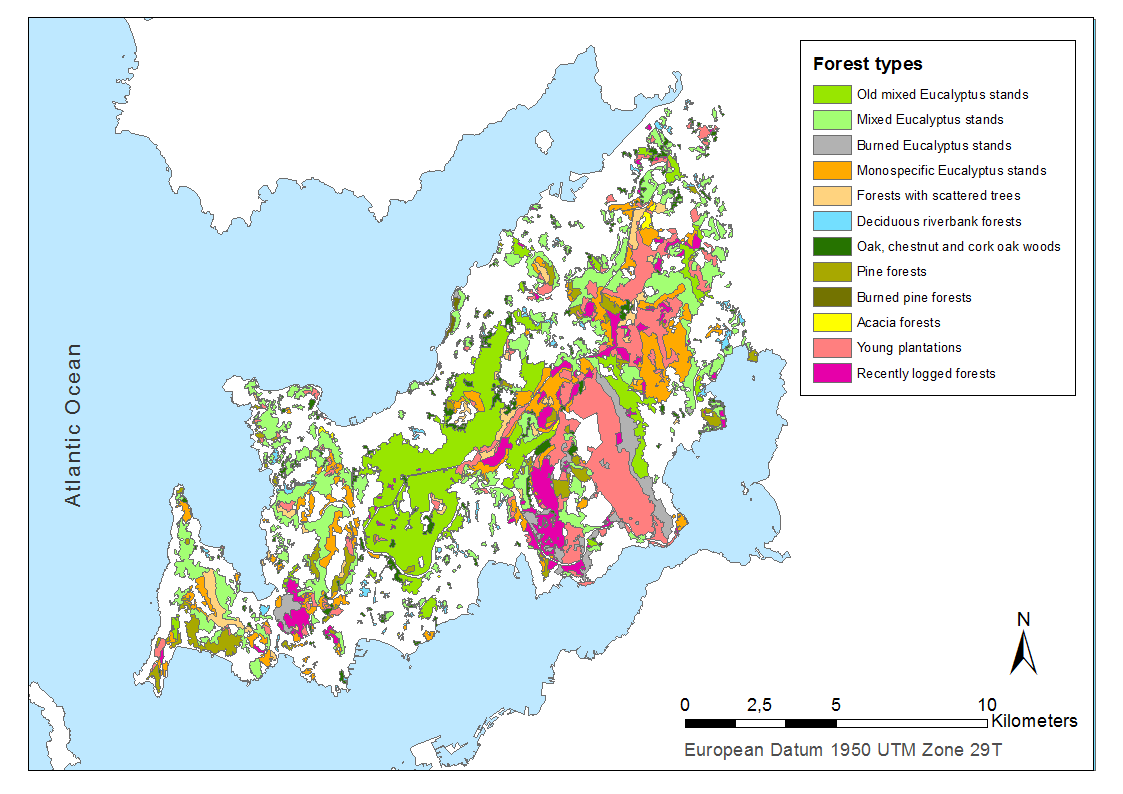


**S2 Figure**. Different forest types on the Morrazo peninsula in northwestern Spain. The figure was prepared based on forest stand composition and structure determined from aerial photographs and confirmed with field visits. White areas are non-forest habitats. Originally drawn by the authors.
