# Supplementary material for: Assessing the ability of novel ecosystems to support animal wildlife through analysis of diurnal raptor territoriality
Source: PLoS One. 2018 Oct 16;13(10):e0205799. doi: 10.1371/journal.pone.0205799 (PMC6191124; doi:10.1371/journal.pone.0205799)
Supplement: S1 Table — Proportion of total forest area occupied by each forest type, relative and average (± SD) tree height in the forest patch, relative and average abundance of eucalyptus trees, large eucalyptus and large oaks. Average tree height and abundance were measured in 10 m radius circular plots (0.03 ha); n = number of sampled plots; DBH = Diameter at breast height. For sample details, see García-Salgado et al. (2018)**. (DOCX) [file pone.0205799.s003.docx]

**Supporting Information**

**Assessing the ability of novel ecosystems to support animal wildlife through analysis of diurnal raptor territoriality**

S. Martínez-Hesterkamp, S. Rebollo, L. Pérez-Camacho, G. García-Salgado and J.M. Fernández-Pereira

**S1 Table**. Characteristics and description of forest types based on composition and structure: proportion of total forest area occupied by each forest type, relative and average (± SD) tree height in the forest patch, relative and average abundance of eucalyptus trees, large eucalyptus and large oaks. Average tree height and abundance were measured in 10 m radius circular plots (0.03 ha); *n* = number of sampled plots; *DBH* = Diameter at breast height. For sample details, see García-Salgado et al. (2018)**.

|  | Forest type* | % Forest Area | *n* | Tree height (m) | Eucalyptus cover  (Trees *DBH*>15 cm 0.03 ha^-1^) | Presence large eucalyptus  (Trees *DBH*>30 cm 0.03 ha^-1^) | Presence large oaks  (Trees *DBH*>30 cm 0.03 ha^-1^) | Description |  |
| --- | --- | --- | --- | --- | --- | --- | --- | --- | --- |
| 1 | Old mixed Eucalyptus stands | 21.0 | 124 | High  (31.8 ± 10.0) | High  (8.0 ± 6.2) | High  (2.2 ± 2.2) | High  (0.5 ± 0.9) | Mixed eucalyptus forest with large eucalyptus and high cover of oak and pine |  |
| 2 | Mixed Eucalyptus stands | 23.9 | 116 | High  (29.4 ± 9.9) | High  (10.5 ± 6.6) | High  (2.6 ± 2.3) | Medium  (0.1 ± 0.5) | Mixed eucalyptus forest with large eucalyptus and medium cover of oak and pine |  |
| 3 | Burned Eucalyptus stands | 4.1 | 9 | High  (23.3 ± 10.5) | High  (6.1 ± 4.5) | High  (1.1 ± 1.8) | Variable  (0) | Mixed or monospecific eucalyptus stands, partially burned with large eucalyptus and variable cover of oak and pine |  |
| 4 | Monospecific Eucalyptus stands | 12.2 | 16 | High  (28.5 ± 20.6) | High  (9.2 ± 6.9) | High  (1.3 ± 2.2) | Low  (0) | Monospecific and contemporary eucalyptus forest |  |
| 5 | Forests with scattered trees | 3.2 | - | Medium | Variable | Variable | Variable | Forest (mainly eucalyptus) with scattered trees |  |
| 6 | Deciduous riverbank forests | 1.7 | - | Medium | Low | Low | Medium | Deciduous riparian forest of alder, willow and oak |  |
| 7 | Oak, chestnut and cork oak woods | 4.7 | 3 | Medium  (22.7 ± 11.0) | Low  (3.3 ± 4.9) | Low  (0.3 ± 0.6) | Variable  (0) | Forest of oak, chestnut or cork oak |  |
| 8 | Pine forests | 5.0 | 4 | Medium  (22.0 ± 10.3) | Low  (3.8 ± 5.7) | Low  (2.0 ± 2.4) | Low  (0) | Pine forest |  |
| 9 | Burned pine forests | 0.2 | - | Medium | Low | Low | Low | Partially burned pine forest |  |
| 10 | Acacia forests | 0.4 | - | Medium | Low | Low | Low | Australian blackwood forest |  |
| 11 | Young plantations | 16.6 | 5 | Low  (15.0 ± 7.9) | High  (5.6 ± 6.8) | Low  (0) | Low  (0) | Young tree plantation mainly of eucalyptus |  |
| 12 | Recently logged forests | 7.1 | - | Low | Variable | Low | Low | Parcel recently harvested without tree vegetation, or only young plantations mainly of eucalyptus |  |
|  | *Listed in order of decreasing complexity of forest composition and structure.  **García-Salgado G, Rebollo S, Pérez-Camacho L, Martínez-Hesterkamp S, De la Montaña E, Domingo-Muñoz R, et al. Breeding habitat preferences and reproductive  success of Northern Goshawk (*Accipiter gentilis*) in exotic Eucalyptus plantations in southwestern Europe. For Ecol Manag. 2018;409:817-25. | | | | | | | | |
