# Supplementary material for: Assessing the ability of novel ecosystems to support animal wildlife through analysis of diurnal raptor territoriality
Source: PLoS One. 2018 Oct 16;13(10):e0205799. doi: 10.1371/journal.pone.0205799 (PMC6191124; doi:10.1371/journal.pone.0205799)
Supplement: S2 Table — Prey species in the Northern Goshawk, Eurasian Sparrowhawk and Common Buzzard diets during the breeding season in the study area. 2,618 prey items of Goshawk were identified from camera images taken at the nests and from uneaten prey remains collected in the nests and surrounding plucking sites during 2008−2011 (77 nests were studied in 29 nesting territories and at least 31 different prey species were hunted). See García-Salgado et al. (2015) and Rebollo et al. (2018) for a complete description of the diet of Goshawk in the area and of the methodology used to study the diet. 1,021 prey items of Sparrowhawk were identified from uneaten prey remains collected at the nests and surrounding plucking sites during 2010–2011 and 2014–2015 (70 nests were studied in 46 nesting territories and at least 51 different prey species were hunted)–unpublished data-. 27 prey items of Buzzard were identified from uneaten prey remains collected at the nests and surrounding plucking sites during 2010–2017 (21 nests were studied in 18 nesting territories and at least 12 different prey species were hunted)–unpublished data-. At least 71 different species of birds, mammals or reptiles were hunted by the three raptor species. (DOCX) [file pone.0205799.s004.docx]

**Supporting Information**

**Assessing the ability of novel ecosystems to support animal wildlife through analysis of diurnal raptor territoriality**

S. Martínez-Hesterkamp, S. Rebollo, L. Pérez-Camacho, G. García-Salgado and J.M. Fernández-Pereira

**S2 Table.** Prey species in the Northern Goshawk, Eurasian Sparrowhawk and Common Buzzard diets during the breeding season in the study area. 2,618 prey items of Goshawk were identified from camera images taken at the nests and from uneaten prey remains collected in the nests and surrounding plucking sites during 2008−2011 (77 nests were studied in 29 nesting territories and at least 31 different prey species were hunted). See García-Salgado et al. (2015) and Rebollo et al. (2018) for a complete description of the diet of Goshawk in the area and of the methodology used to study the diet. 1,021 prey items of Sparrowhawk were identified from uneaten prey remains collected at the nests and surrounding plucking sites during 2010-2011 and 2014-2015 (70 nests were studied in 46 nesting territories and at least 51 different prey species were hunted) –unpublished data-. 27 prey items of Buzzard were identified from uneaten prey remains collected at the nests and surrounding plucking sites during 2010-2017 (21 nests were studied in 18 nesting territories and at least 12 different prey species were hunted) –unpublished data-. At least 71 different species of birds, mammals or reptiles were hunted by the three raptor species.

| **BIRDS** | **Species/taxonomic group** | **Northern Goshawk** | **Eurasian Sparrowhawk** | **Common Buzzard** |
| --- | --- | --- | --- | --- |
| Black-Headed Gull | *Chroicocephalus ridibundus* |  | **x** |  |
| Black Redstart | *Phoenicurus ochruros* |  | **x** |  |
| Budgerigar | *Melopsittacus undulatus* |  | **x** |  |
| Carrion Crow | *Corvus corone* | **x** |  |  |
| Cirl Bunting | *Emberiza cirlus* |  | **x** |  |
| Coal Tit | *Periparus ater* |  | **x** |  |
| Common Blackbird | *Turdus merula* | **x** | **x** | **x** |
| Common Chaffinch | *Fringilla coelebs* |  | **x** |  |
| Common Chiffchaff | *Phylloscopus collybita* |  | **x** |  |
| Common Cuckoo | *Cuculus canorus* | **x** | **x** |  |
| Common Linnet | *Carduelis cannabina* |  | **x** |  |
| Common Magpie | *Pica pica* |  | **x** |  |
| Common Quail | *Coturnix coturnix* | **x** | **x** |  |
| Common Wood Pigeon | *Columba palumbus* | **x** | **x** | **x** |
| Dartford Warbler | *Sylvia undata* |  | **x** |  |
| Domestic Chicken | *Gallus gallus domesticus* | **x** |  | **x** |
| Dunnock | *Prunella modularis* |  | **x** |  |
| Eurasian Blackcap | *Sylvia atricapilla* |  | **x** |  |
| Eurasian Blue Tit | *Cyanistes caeruleus* |  | **x** |  |
| Eurasian Bullfinch | *Pyrrhula pyrrhula* |  | **x** |  |
| Eurasian Collared Dove | *Streptopelia decaocto* | **x** | **x** | **x** |
| Eurasian Golden Oriole | *Oriolus oriolus* | **x** |  |  |
| Eurasian Jay | *Garrulus glandarius* | **x** | **x** |  |
| Eurasian Magpie | *Pica pica* | **x** |  |  |
| Eurasian Sparrowhawk | *Accipiter nisus* | **x** |  |  |
| Eurasian Tree Sparrow | *Passer montanus* |  | **x** |  |
| European Crested Tit | *Lophophanes cristatus* |  | **x** |  |
| European Green Woodpecker | *Picus viridis* | **x** | **x** |  |
| European Greenfinch | *Chloris chloris* |  | **x** |  |
| European Nightjar | *Caprimulgus europaeus* | **x** | **x** |  |
| European Pied Flycatcher | *Ficedula hypoleuca* |  | **x** |  |
| European Robin | *Erithacus rubecula* |  | **x** |  |
| European Serin | *Serinus serinus* |  | **x** |  |
| European Stonechat | *Saxicola rubicola* |  | **x** |  |
| European Turtle Dove | *Streptopelia turtur* | **x** | **x** |  |
| Feral Pigeon | *Columba livia f. domestica* | **x** | **x** | **x** |
| Great Spotted Woodpecker | *Dendrocopos major* | **x** | **x** |  |
| Great Tit | *Parus major* |  | **x** |  |
| Hawfinch | *Coccothraustes coccothraustes* |  | **x** |  |
| Hoopoe | *Upupa epops* |  | **x** |  |
| House Sparrow | *Passer domesticus* |  | **x** |  |
| Long-Tailed Tit | *Aegithalos caudatus* |  | **x** |  |
| Meadow Pipit | *Anthus pratensis* |  | **x** |  |
| Mistle Thrush | *Turdus viscivorus* | **x** | **x** |  |
| Rock Bunting | *Emberiza cia* |  | **x** |  |
| Sardinian Warbler | *Sylvia melanocephala* |  | **x** |  |
| Sedge Warbler | *Acrocephalus schoenobaenus* |  | **x** |  |
| Short-Toed Treecreeper | *Certhia brachydactyla* |  | **x** |  |
| Song Thrush | *Turdus philomelos* | **x** | **x** |  |
| Spotless Starling | *Sturnus unicolor* | **x** | **x** |  |
| Tawny Owl | *Strix aluco* | **x** |  |  |
| White Wagtail | *Motacilla alba* |  | **x** |  |
| Winter Wren | *Troglodytes troglodytes* |  | **x** |  |
| Yellow-legged Gull | *Larus michahellis* | **x** |  |  |
| Zitting Cisticola | *Cisticola juncidis* |  | **x** |  |
| Unidentified duck | *Anatidae sp.* |  | **x** |  |
| Unidentified parrot | *Psittacidae sp.* | **x** |  |  |
| Unidentified wader | *Scolopacidae sp.* |  | **x** |  |
|  |  |  |  |  |
| **MAMMALS** |  |  |  |  |
| American Mink | *Neovison vison* | **x** |  |  |
| European Mole | *Talpa europaea* | **x** |  | **x** |
| European Rabbit | *Oryctolagus cuniculus* | **x** |  | **x** |
| Least Weasel | *Mustela nivalis* | **x** |  |  |
| Red Squirrel | *Sciurus vulgaris* | **x** |  |  |
| West European Hedgehog | *Erinaceus europaeus* | **x** |  |  |
| Wood Mouse | *Apodemus sylvaticus* |  | **x** | **x** |
| Unidentified micromammal |  | **x** |  |  |
| Unidentified rat | *Rattus sp.* | **x** |  |  |
| Unidentified shrew | *Soricidae sp.* |  |  | **x** |
|  |  |  |  |  |
| **REPTILES** |  |  |  |  |
| Ocellated Lizard | *Timon lepidus* | **x** |  | **x** |
| Slow Worm | *Anguis fragilis* |  |  | **x** |
| Unidentified snake | *Colubridae spp.* |  |  | **x** |
