# Supplementary material for: Assessing the ability of novel ecosystems to support animal wildlife through analysis of diurnal raptor territoriality
Source: PLoS One. 2018 Oct 16;13(10):e0205799. doi: 10.1371/journal.pone.0205799 (PMC6191124; doi:10.1371/journal.pone.0205799)
Supplement: S3 Table — Country, population, time scale estimation, number of nests, mean nearest neighbor distances (NND), SD of the mean, pair density, and data sources for all populations of Goshawk, Sparrowhawk and Buzzard used to prepare Fig 2. (DOCX) [file pone.0205799.s005.docx]

**Supporting Information**

**Assessing the ability of novel ecosystems to support animal wildlife through analysis of diurnal raptor territoriality**

S. Martínez-Hesterkamp, S. Rebollo, L. Pérez-Camacho, G. García-Salgado and J.M. Fernández-Pereira

**S3 Table.** Country, population, time scale estimation, number of nests, mean nearest neighbor distances (NND), SD of the mean, pair density, and data sources for all populations of Goshawk, Sparrowhawk and Buzzard used to prepare Figure 2.

| **Species** | **Country** | **Population** | **Estimation^1^** | **No. of Nests** | **Mean NND^2^** | **SD** | **Pairs Density** | **Source** |
| --- | --- | --- | --- | --- | --- | --- | --- | --- |
| Northern Goshawk | Poland | Coniferous forest | Anual | 11 | 2257.3 | 572,5 | 12.4 | [[1](#_ENREF_1)] |
|  | Poland | Lasy P Forest | Anual | 21 | 3070 | 1909.2 | 12.7 | [[2](#_ENREF_2)] |
|  | Poland | Siedlce | Anual | 5 | 3460 |  | 5.4 | [[3](#_ENREF_3)] |
|  | Germany | Niederrheinische Bucht-All | Anual | 87 | 2210 | 720,0 | 2.6 | [[4](#_ENREF_4)] |
|  | Italy | Central Italy | Anual | 16 | 3500 | 1020 | 5.03 | [[5](#_ENREF_5)] |
|  | France | Eastern France | Multi-annual | 126 | 2840 | 580 | 6.3 | [[6](#_ENREF_6)] |
|  | France | Northern France | Anual | 19 | 2750 | 580 | 2.7 | [[7](#_ENREF_7)] |
|  | Several | Europe South | Anual | N/A | 3400 |  | 5 | [[8](#_ENREF_8)] |
|  | Norway | S Norway | Multi-annual | 24.6 | 4600 |  | 4.54 | [[9](#_ENREF_9)] |
|  | Finland | Espoo | Multi-annual | 20 | 4000 | 1400 | 5 | [[10](#_ENREF_10)] |
|  | Finland | Hyvinkaa-Askola | Multi-annual | 31 | 3400 | 900 | 7 | [[10](#_ENREF_10)] |
|  | Finland | Sipoo | Multi-annual | 23 | 3200 | 900 | 7 | [[10](#_ENREF_10)] |
|  | Japan | Central Japan | Multi-annual | 37 | 1740 | 590 | 8.08 | [[11](#_ENREF_11)] |
|  | Sweden | Grimso | Multi-annual | 6.5 | 6300 |  | 3 | [[12](#_ENREF_12)] |
|  | Sweden | Ramnas | Anual | 7 | 6400 |  | N/A | [[12](#_ENREF_12)] |
|  | Poland | Wigry National Park | Multi-annual | 9.5 | 3689 | 1192 | 6.3 | [[13](#_ENREF_13)] |
|  | Spain | Morrazo | Anual | 20.2 | 2234.1 | 705.3 | 10.4 | This study |
|  | Spain | Morrazo | Multi-annual | 29 | 1933.3 | 454.4 | 15.8 | This study |

S3 Table. Continuation.

| **Species** | **Country** | **Population** | **Estimation^1^** | **No. of Nests** | **Mean NND^2^** | **SD** | **Pairs Density^3^** | **Source** |
| --- | --- | --- | --- | --- | --- | --- | --- | --- |
| Eurasian Sparrowhawk | Scotland | Lochar | Anual | 5 | 700 | 200 | 312.5 | [[14](#_ENREF_14)] |
|  | Britain | Windsor | Multi-annual | 46 | 580 | 180 | N/A | [[15](#_ENREF_15)] |
|  | Britain | Ae Forest | Multi-annual | 29 | 1550 | 640 | N/A | [[15](#_ENREF_15)] |
|  | Britain | Annandale | Multi-annual | 121 | 600 | 90 | N/A | [[15](#_ENREF_15)] |
|  | Britain | Clashindarroch Forest | Multi-annual | 13 | 1280 | 150 | N/A | [[15](#_ENREF_15)] |
|  | Britain | Eskdale | Multi-annual | 50 | 620 | 140 | N/A | [[15](#_ENREF_15)] |
|  | Britain | Forres | Multi-annual | 57 | 610 | 260 | N/A | [[15](#_ENREF_15)] |
|  | Britain | Kielder Forest | Multi-annual | 8 | 1900 | 530 | N/A | [[15](#_ENREF_15)] |
|  | Britain | Lower Deeside | Multi-annual | 16 | 910 | 180 | N/A | [[15](#_ENREF_15)] |
|  | Britain | Lowland | Multi-annual | 13 | 750 | 370 | N/A | [[15](#_ENREF_15)] |
|  | Britain | Mar Forest | Multi-annual | 9 | 2060 | 290 | N/A | [[15](#_ENREF_15)] |
|  | Britain | Mid-Deeside | Multi-annual | 36 | 1120 | 220 | N/A | [[15](#_ENREF_15)] |
|  | Britain | Slaley | Multi-annual | 48 | 890 | 280 | N/A | [[15](#_ENREF_15)] |
|  | Britain | South Solway Plain | Multi-annual | 20 | 460 | 90 | N/A | [[15](#_ENREF_15)] |
|  | Britain | Speyside | Multi-annual | 42 | 2030 | 330 | N/A | [[15](#_ENREF_15)] |
|  | Britain | Tregaron | Multi-annual | 22 | 1230 | 160 | N/A | [[15](#_ENREF_15)] |
|  | Britain | Upland | Multi-annual | 12 | 890 | 190 | N/A | [[15](#_ENREF_15)] |
|  | Britain | Upper Deeside | Multi-annual | 9 | 1390 | 180 | N/A | [[15](#_ENREF_15)] |
|  | Britain | Wytham | Multi-annual | 7 | 760 |  | N/A | [[15](#_ENREF_15)] |
|  | Denmark | Sindal | Anual | 296 | 1400 | 840 | 22.1 | [[16](#_ENREF_16)] |
|  | Denmark | Vest | Anual | 662 | 2000 | 1040 | 7.3 | [[16](#_ENREF_16)] |
|  | Norway | S Norway | Multi-annual | 43.5 | 1875 | 250 | N/A | [[17](#_ENREF_17)] |
|  | Finland | Askola | Multi-annual | 11 | 2100 | 600 | 9 | [[10](#_ENREF_10)] |
|  | Finland | Espoo | Multi-annual | 21 | 2200 | 700 | 11 | [[10](#_ENREF_10)] |
|  | Finland | Sipoo | Multi-annual | 35 | 2000 | 500 | 9 | [[10](#_ENREF_10)] |
|  | Britain | Rockingham | Anual | 39.3 | 737 | 344 | 21.88 | [[18](#_ENREF_18)] |
|  | Spain | Morrazo | Anual | 29.6 | 1567.8 | 726.3 | 13.6 | This study |
|  | Spain | Morrazo | Multi-annual | 57 | 1052.5 | 421.8 | 31.1 | This study |

S3 Table. Continuation.

| **Species** | **Country** | **Population** | **Estimation^1^** | **No. of Nests** | **Mean NND^2^** | **SD** | **Pairs Density** | **Source** |
| --- | --- | --- | --- | --- | --- | --- | --- | --- |
| Common Buzzard | Poland | Coniferous forest | Anual | 18 | 1433.8 | 795.7 | 20.3 | [[1](#_ENREF_1)] |
|  | Poland | Oak-hornbeam wood | Anual | 15 | 817.3 | 260.3 | 85.2 | [[1](#_ENREF_1)] |
|  | Poland | Lasy P Forest | Anual | 131 | 852.5 | 266 | 78 | [[2](#_ENREF_2)] |
|  | Wales | Dartmoor, Devon | Multi-annual | 16 | 1000 | 500 | 48 | [[19](#_ENREF_19)] |
|  | Wales | Migneint-Hiraethog | Multi-annual | 62 | 1530 | 580 | 14.1 | [[19](#_ENREF_19)] |
|  | Wales | Snowdonia | Multi-annual | 96 | 1950 | 750 | 10.3 | [[19](#_ENREF_19)] |
|  | Wales | Speyside | Multi-annual | 38 | 1500 |  | 22 | [[19](#_ENREF_19)] |
|  | Poland | Siedlce | Anual | 33 | 1556 |  | 25.4 | [[3](#_ENREF_3)] |
|  | Scotland | Langholm | Anual | 16 | 1900 | 970 | 7.6 | [[20](#_ENREF_20)] |
|  | Luxembourg | Ost-Luxemburg | Anual | 29 | 590 |  | 78.4 | [[21](#_ENREF_21)] |
|  | Germany | Niederrheinische Bucht-Börde-area | Anual | 121 | 720 | 530 | 10.4 | [[4](#_ENREF_4)] |
|  | Germany | Niederrheinische Bucht-Ville-area | Anual | 139 | 1130 | 550 | 10.4 | [[4](#_ENREF_4)] |
|  | Germany | Eastern Westphalia | Anual | 106 | 1406.1 | 495.1 | 14.7 | [[22](#_ENREF_22)] |
|  | Britain | Walles-Farmland | Anual | 238 | 870 | 30 | 41 | [[23](#_ENREF_23)] |
|  | Britain | Walles-Upland | Anual | 322 | 1130 | 40 | 24 | [[23](#_ENREF_23)] |
|  | Italy | Central Italy | Multi-annual | 32 | 2500 | 540 | 8.3 | [[24](#_ENREF_24)] |
|  | Spain | Tenerife-Teno | Anual | 31 | 1081 | 547 | 22.14 | [[25](#_ENREF_25)] |
|  | Italy | Italian pre-Alps | Anual | 108 | 1108 |  | 29 | [[26](#_ENREF_26)] |
|  | Britain | West Midlands SO37 | Multi-annual | 70 | 830 |  | 71 | [[27](#_ENREF_27)] |
|  | Britain | West Midlands SO77 | Multi-annual | 12 | 1330 |  | 20 | [[27](#_ENREF_27)] |
|  | Finland | Askola | Multi-annual | 21 | 2000 | 400 | 18 | [[10](#_ENREF_10)] |
|  | Finland | Sipoo | Multi-annual | 24 | 2700 | 600 | 9 | [[10](#_ENREF_10)] |
|  | Britain | Dorset Coast | Anual | 119 | 1540 | 180 | N/A | [[28](#_ENREF_28)] |
|  | Spain | Bizkaia | Multi-annual | 18 | 1254 |  | 45 | [[29](#_ENREF_29)] |
|  | Spain | Murcia | Multi-annual | 7.5 | 2123 | 466.7 | 7.5 | [[29](#_ENREF_29)] |
|  | Spain | Morrazo | Anual | 38.2 | 1323.7 | 775.5 | 17.7 | This study |
|  | Spain | Morrazo | Multi-annual | 84 | 742.3 | 355.1 | 45.8 | This study |

Note: ^1^ Estimation method: NNDs estimated annually (active nests) or multi-annually (nesting territories over the entire study period). ^2^ Mean NNDs (m). ^3^ Density of breeding pairs (pairs/100 Km^2^).

**References**

1. Bielański W. Nesting preferences of Common Buzzard *Buteo buteo* and Goshawk *Accipiter gentilis* in forest stands of different structure (Niepolomice Forest, Southern Poland). Biologia. 2006;61(5):597-603. PubMed PMID: WOS:000243710100022.

2. Buczek T, Keller M, Rozycki AL. Legowe ptaki szponiaste Falconiformes lasow Parczzewskich - zmiany liczebnosci i rozmieszczenia w latach 1991-1993 i 2002 - 2004. [Breeding birds of prey Falconiformes in the Lasy Parczewskie forest - fluctuations in numbers and changes in distribution in the spans of 1991-1993 and 2002-2004.]. Notatki Ornitol. 2007;48(4):217-31.

3. Dombrowski A, Golawski A, Szymkiewicz M. Gniazdowanie ptakÃ³w drapieznych Falconiformes i kruka Corvus corax w krajobrazie rolniczym pod Siedlcami w latach 1978 i 1999. [Breeding birds of prey Falconiformes and the Raven Corvus corax in the agricultural landscape near Siedlce in 1978 and 1999.] (English). Notatki Ornitol. 2000;41(3):201-12.

4. Kostrzewa A. Interspecific interference competition in three European raptor species. Ethol Ecol Evol. 1991;3(2):127-43. PubMed PMID: ISI:A1991FQ30800004.

5. Penteriani V. Long-term study of a Goshawk breeding population on a Mediterranean mountain (Abruzzi apennines, central Italy): Density, breeding performance and diet. J Raptor Res. 1997;31(4):308-12. PubMed PMID: ISI:000071709100002.

6. Penteriani V, Faivre B, Frochot B. An approach to identify factors and levels of nesting habitat selection: a cross-scale analysis of Goshawk preferences. Ornis Fenn. 2001;78(4):159-67. PubMed PMID: WOS:000173396200002.

7. Penteriani V, Mathiaut M, Boisson G. Immediate species responses to catastrophic natural disturbances: Windthrow effects on density, productivity, nesting stand choice, and fidelity in Northern Goshawks (*Accipiter gentilis*). Auk. 2002;119(4):1132-7. PubMed PMID: ISI:000180649200023.

8. Rutz C, Bijlsma RG, Marquiss M, Kenward R. Population limitation in the Northern Goshawk in Europe: a review with case studies. Stud Avian Biol. 2006;31:158-97.

9. Selas V. Influence of prey availability on re-establishment of Goshawk *Accipiter gentilis* nesting territories. Ornis Fenn. 1997;74(3):113-20. PubMed PMID: ISI:A1997XX37900001.

10. Solonen T. Spacing of birds of prey in Southern Finland. Ornis Fenn. 1993;70(3):129-43. PubMed PMID: ISI:A1993MB14500001.

11. Uchida H, Takayanagi S, Suzuki S, Watanabe T, Ishimatu Y, Tanaka I, et al. Breeding ecology of Northern Goshawk at hilly terrain area in central Japan. Jpn J Ornithol. 2007;56(2):131-40.

12. Widen P. Breeding and movements of Goshawks in boreal forests in Sweden. Holarct Ecol. 1985;8(4):273-9.

13. Zawadzka D, Zawadzki J. The Goshawk *Accipiter gentilis* in Wigry National Park (NE Poland)- numbers, breeding results, diet composition and prey selection. Acta Ornithol. 1998;33(3/4):181-90.

14. Marquiss M, Newton I. A radio-tracking study of the ranging behavior and dispersion of European Sparrowhawks *Accipiter nisus*. J Anim Ecol. 1982;51(1):111-33. PubMed PMID: WOS:A1982NC93600009.

15. Newton I, Wyllie I, Mearns R. Spacing of Sparrowhawks in relation to food-supply. J Anim Ecol. 1986;55(1):361-70. PubMed PMID: WOS:A1986AYM0800025.

16. Nielsen JT. Spurvehogens *Accipiter nisus* bestandsudvikling, ynglehabitat, alderssammensaetning og ungeproduktion i Vendsyssel, 1977-97. Dan Ornitol Foren Tidsskr. 2004;98(4):147-62.

17. Selas V. Breeding density of Sparrowhawk *Accipiter nisus* in relation to nest site availability, hatching success and winter weather. Ornis Fenn. 1997;74(3):121-9. PubMed PMID: WOS:A1997XX37900002.

18. Wyllie I, Newton I. Demography of an increasing population of Sparrowhawks. J Anim Ecol. 1991;60(3):749-66.

19. Dare PJ, Barry JT. Population size, density and regularity in nest spacing of Buzzards *Buteo buteo* in two upland regions of North Wales. Bird Study. 1990;37:23-9. PubMed PMID: WOS:A1990CW03200005.

20. Graham IM, Redpath SM, Thirgood SJ. The diet and breeding density of Common Buzzards *Buteo buteo* in relation to indexes of prey abundance. Bird Study. 1995;42:165-73. PubMed PMID: WOS:A1995RG95100008.

21. Kiefer J. Der Maüsebussard *Buteo buteo* in Ost-Luxemburg. Regulus - Wissenschaftliche Berichte. 2006;21:31-7.

22. Kruger O. The importance of competition, food, habitat, weather and phenotype for the reproduction of Buzzard *Buteo buteo*. Bird Study. 2004;51:125-32. PubMed PMID: ISI:000222597600005.

23. Newton I, Davis PE, Davis JE. Ravens and Buzzards in relation to sheep farming and forestry in Wales. J Appl Ecol. 1982;19(3):681-706.

24. Penteriani V, Faivre B. Breeding density and landscape-level habitat selection of Common Buzzards (*Buteo buteo*) in a mountain area (Abruzzo Apennines, Italy). J Raptor Res. 1997;31(3):208-12. PubMed PMID: ISI:A1997XY96400002.

25. Rodríguez B, Siverio F, Rodríguez A, Siverio M, Hernández JJ, Figuerola J. Density, habitat selection and breeding biology of Common Buzzards *Buteo buteo* in an insular environment. Bird Study. 2010;57(1):75-83. PubMed PMID: WOS:000274423800008.

26. Sergio F, Boto A, Scandolara C, Bogliani G. Density, nest sites, diet, and productivity of Common Buzzards (*Buteo buteo*) in the Italian pre-Alps. J Raptor Res. 2002;36(1):24-32. PubMed PMID: ISI:000174912500005.

27. Sim IMW, Cross AV, Lamacraft DL, Pain DJ. Correlates of Common Buzzard *Buteo buteo* density and breeding success in the West Midlands. Bird Study. 2001;48:317-29. PubMed PMID: ISI:000172101700006.

28. Walls SS, Kenward RE. Spatial consequences of relatedness and age in Buzzards. Anim Behav. 2001;61:1069-78. PubMed PMID: ISI:000169734200004.

29. Zuberogoitia I, Martinez JE, Martinez JA, Zabala J, Calvo JF, Castillo I, et al. Influence of management practices on nest site habitat selection, breeding and diet of the Common Buzzard *Buteo buteo* in two different areas of Spain. Ardeola. 2006;53(1):83-98. PubMed PMID: ISI:000240246400007.
